# Establishing Core Competencies and a Professional Curriculum for the Care Service Department in Vocational High Schools in Taiwan

**DOI:** 10.3390/ijerph19021009

**Published:** 2022-01-17

**Authors:** Nan-Chen Hsieh, Shu-Fang Vivienne Wu, Juin-Ming Tsai, Li-Ju Lin, Juo-Hsiang Sun

**Affiliations:** 1 Department of Information Management, National Taipei University of Nursing and Health Sciences, Taipei City 112, Taiwan; nchsieh@ntunhs.edu.tw; 2 Research Center for Healthcare Industry Innovation, National Taipei University of Nursing and Health Sciences, Taipei City 112, Taiwan; shufang@ntunhs.edu.tw; 3 School of Nursing, National Taipei University of Nursing and Health Sciences, Taipei City 112, Taiwan; 4 Department of Gerontological Health Care, National Taipei University of Nursing and Health Sciences, Taipei City 112, Taiwan; juinming@ntunhs.edu.tw; 5 International and Cross-Strait Education Center, National Taipei University of Nursing and Health Sciences, Taipei City 112, Taiwan; liju@ntunhs.edu.tw; 6 Department of Long-Term Care, National Taipei University of Nursing and Health Sciences, Taipei City 112, Taiwan

**Keywords:** vocational high school, care service department, core competencies, professional curriculum, long-term care education

## Abstract

In response to the emergence of the aging society, the vocational high school education system in Taiwan has established a care service department since 2018. The purpose of this study was to develop core competencies and a professional curriculum for the care service department in vocational high schools. First, this study invited 20 experts and scholars to take part in a focus group to collect suggestions as the basis for the development of core competencies and a curriculum. Second, this study invited 10 experts and scholars to participate in three rounds of a Delphi survey to evaluate the planning for the development of core competencies and a curriculum that meet educational needs. In this study, we identified eight core competency constructs and 15 indicators across two dimensions relating to the care services taught in vocational high schools. We then designed 26 professional subjects according to the core competencies. We identified the core competencies for long-term care service education and devised a professional curriculum to foster the skills and knowledge among students that are required for successfully meeting the care needs of a rapidly aging society through work in the long-term care industry after graduation.

## 1. Introduction

### 1.1. Context of Study

Changes in the demographic structure in Taiwan, specifically an aging population and a declining birth rate, are causing concern among policymakers. Based on the population statistics released by the National Development Council in August 2018, Taiwan is estimated to become a super-aged society by 2026, whereby the proportion of older adults aged 65 years and older is expected to increase from 14.5% to 20%. Thereafter, the senior population is estimated to continue to grow, reaching 41.2% by 2065 [1]. This growth is expected to be accompanied by an increased demand for long-term care. Therefore, long-term elderly care is a public concern that must be addressed. The 2017 Report of the Senior Citizen Condition Survey indicated that 41% of adults between 55 and 64 years of age reported having a chronic disease. This percentage increased to 65% for older adults aged 65 and older (over half the respondents). In this age group, approximately 30% of the older adults experienced difficulty in performing at least one activity of daily living (ADL) or instrumental activity of daily living (IADLs) [2]. In 2019, the population requiring long-term care and assistance was 907,000 and may soon exceed one million. As the health conditions of older adults change, the demand for long-term care and caregivers will gradually increase. To solve the problem, the government in Taiwan has not only begun to promote occupational education for members of society but also hopes that young people will dedicate themselves to the long-term care industry. Therefore, long-term care has been viewed as a professional subject. Specialized departments have been established in universities and vocational high schools to cultivate long-term care personnel with a view to making care service personnel younger and preparing them to cope with the huge needs for care in the future. The purpose of this study was to establish core professional competencies for the care service departments of vocational high schools and develop a corresponding professional curriculum.

### 1.2. Literature Review

#### 1.2.1. Promote Long-Term Caregiver Cultivation

In response to the rapidly growing demand for care, the Ten-Year Plan for Long-Term Care in Taiwan was launched in 2007 to establish a long-term care service system, strengthen the infrastructure for the provision of long-term care services, ensure that older adults and people with physical or mental disabilities receive adequate care, and enhance people’s ability to live independently with improved quality of life while maintaining their dignity and autonomy [3]. The government further announced the Long-Term Care Services Act and launched the Ten-Year Plan for Long-Term Care in Taiwan 2.0 in June 2015. Under these initiatives, the central government, local governments, and private organizations collaborate in streamlining long-term care development and building a comprehensive, community-centered, diversified, continuous, and universal long-term care service system that encompasses family care, home care, community care, and residential/institutional care, thereby fulfilling the goal of allowing older adults to age in place. 

In addition to building a robust long-term care system, increasing the pool of long-term caregivers is key to establishing a long-term care service delivery system. The lack of caregivers signifies an inability to meet care demands. The Ministry of Health and Welfare generated an inventory of long-term care resources in Taiwan in 2004 and found a severe shortage of caregivers in Taiwan [4]. During this period, Taiwan had 26,942 caregivers, 30,912 less than the required number of providers. These statistics highlighted the urgent need for a long-term care service system to satisfy the demand for caregivers. In response, the Taiwanese government established the Long-Term Care System Promotion Task Force concurrently with the launch of Long-Term Care 1.0 in 2007 and listed talent cultivation as one of six major objectives. In addition, the Ministry of Education introduced a training program for long-term care professionals. The key objectives of this program are as follows: (1) to provide caregiver training; (2) to ensure satisfactory work conditions and minimize turnover; (3) to encourage professionals to pursue accreditation and enhance their professional image; (4) to educate long-term caregivers through nurturing; (5) to subsidize social work, nursing, occupational therapy, and physiotherapy departments and launch cross-disciplinary long-term care courses; (6) to assist universities and colleges in improving the quality of their long-term care departments; and (7) to continue to provide training to caregivers working in long-term care centers [5].

#### 1.2.2. The Professionalization of Long-Term Care Education

Long-term caregivers are not paid due respect for the work they perform, and their professional role is seldom recognized, resulting in a continuous shortage of caregivers in Taiwan. Many young adults are unwilling to become long-term caregivers because they believe that the public generally considers the industry to be socially and economically inferior to others. They believe that “care” is nonprofessional and offers no potential for future career development. Therefore, only five universities in Taiwan offered long-term care as a major in 2005, cumulatively enrolling 882 students [6]. To attract young adults to long-term care, the government began to focus on improving the professionalism and value of instruction in long-term care offered at the university level. In 2015, the Ministry of Education established the Health Care Industry–University Cooperation Center at National Taipei University of Nursing and Health Sciences. The center serves to promote long-term caregiver cultivation and the professionalization of long-term care education. The center offers a four-module course (caregiver module, home care supervision module, care manager module, and business management module) centered on developing a career in long-term care and internship opportunities to minimize the gap between academic learning and real-world practice. As of 2019, the number of Taiwanese universities offering long-term care as a major had increased to 43, collectively enrolling 7383 students [6]. These results highlight the effectiveness of promoting long-term care education in universities.

However, the care service profession in Taiwan currently only has one single type of care service personnel certificate; before 2018, both trainees from the 90-h vocational training for the general public and graduates from university departments of long-term care could both receive the same professional certificate after taking the exam and engaging with care service jobs, which greatly reduced the intentions of high school graduates to enter departments of long-term care. Therefore, the government in Taiwan took the professional long-term care training curricula in Japan and Australia as reference and found that integral and professional systems of caretakers were established both in Australia and Japan to varying degrees, and the differences in hours and content are linked with with the professional certificates received. Taking Japan for example, the professionalization of long-term care is respected; long-term care personnel in Japan are categorized into four degrees, life support adviser, care staff novice’s trainee, welfare Nakura series, and care manager [7]; meanwhile, the Japan National Council of Social Welfare has introduced a qualification for promotion for care service personnel, and lists the categories of educational training and the objectives of competence development. In addition, there are integral culturation curricula for gerontological care and related certificates in Australia, including four course modules: Certification III and IV in aged care, dementia care, and hospice care. The Certificate III in Individual Support has replaced the previous three certificates: Certificate of Gerontological Care, Certificate III in Home and Community Care and in Disability [8], and Certificate IV in Ageing Support [9]. With professional certificates and integral promotion paths, both Japan and Australia provide new spaces for the care profession, enabling people with practical culturation experiences (occupational training) and formal educational training (high schools and universities, etc.) to take different paths to acquire professional certificates of different degrees, and talent will be more willing to stay in the long-term care industry. As a result, in order to tackle the problem of lack of degree differentiation for long-term care in Taiwan, the government in Taiwan took the culturation system in Japan and Australia as reference in order to differentiate the degree of certificates and qualifications.

To begin with, the Ministry of Education expanded its efforts in developing caregivers by introducing a pilot program for establishing care service departments in vocational high schools at the beginning of 2018. This program encourages vocational high schools in Taiwan to establish care service departments. It aims to create streamlined talent cultivation pathways from high school to university, thereby providing young adults with an attractive route for academic progression and overturning the stereotypes of long-term care and starting to try to classify long-term care, allowing high school graduates and graduates of 90-h vocational training to participate in frontline long-term care jobs, while graduates of university departments of long-term care can further serve as home care supervisors, case managers, and care managers. Care service departments were established in seven schools in 2018, 17 in 2019, and 19 in 2020. The number of applications has risen concurrently with the urgent needs of the long-term care industry in Taiwan. Because the establishment of care service departments was in the experimental stage during the promotion of the 2018 pilot program, no standardized curriculum was available, leading to vocational high schools offering various courses that failed to encompass the professional and career requirements of long-term care. Some subjects were overly difficult and inappropriate for high school students. In response, the K-12 Education Administration, Ministry of Education, entrusted the researchers of this study to highlight the core competencies for care service workers and develop a professional curriculum for care service education in high schools. Vocational high schools aim to equip students with the knowledge required in their professional field, improve their professional knowledge, and help them form a theoretical foundation [10]. In Taiwan, we have moved into the era of 12-Year Compulsory Education since 2019; compulsory education has extended from 9 years to 12 years with a view to elevating the quality of national education and national competency. The 12-Year Compulsory Education includes 9-Year National Education and 3-Year High School Education. High School Education includes two categories: senior high schools and vocational high schools. Teenagers will select their path at the age of 16 according to their specialty. Generally speaking, graduates of senior high schools will enter college for further study, while graduates of vocational high schools will choose to become employed or look for further study. Therefore, vocational high schools will put more emphasis on the employability of students, helping students to acquire national certificates after graduation in order to become employed. Core competencies refer to the abilities and knowledge that should be attained before graduation to succeed in the selected profession or career. They also represent the focal abilities, knowledge, techniques, judgment, attitudes, values, and personalities required for coping with the future environment, society, and career. They include explicit knowledge, techniques, and attitudes [11,12,13]. Vocational high schools provide demand-oriented education to help students secure employment or continue fostering core competencies. The findings of the Preliminary Research on Curriculum Development for Higher Vocational Education indicated that fostering high-quality workers by enhancing their core competencies is key to improving workplace competitiveness. This finding is also the basis for engaging in professional work [14]. These goals are the key objectives that are considered in the development of a professional curriculum for the care service departments of vocational high schools. Therefore, establishing core competencies associated with care services for vocational high schools can help students develop professional competencies and affects the development of professional curricula. In summary, the purpose of this study was to establish core professional competencies for the care service departments of vocational high schools and develop a corresponding professional curriculum. We determined the core competencies that vocational high school students studying care services should possess by the time of high school graduation to meet market requirements. We also identified professional coursework corresponding to each core competency. In this study, we collaborated with several scholars and practitioners specializing in long-term care to design an educational outline for teaching care services in vocational high schools in Taiwan. The outline can serve as a reference for designing professional curricula.

## 2. Methods

### 2.1. Participants

We adopted a qualitative research design and selected the research subjects through purposive sampling. The subjects were scholars and practitioners of long-term care in Taiwan. Research was conducted in two stages. In the first stage, we organized a focus group to collect data. We invited 10 professors and 10 practitioners to participate in a focus group interview. The inclusion criteria were as follows: (1) professors teaching long-term care in Taiwan and promoting the professionalization of long-term care education in Taiwan and (2) practitioners serving in a managerial position in the long-term care industry (care institutions or nonprofit organizations). In the second stage, we adopted the Delphi method to identify the core competencies and develop a professional curriculum that meets teaching requirements. Ten influential and pioneering experts and scholars from the field of long-term care were invited to form an expert panel. In order to make the participants better understand the goal of research and comply with research ethics, we will send descriptions of the research first via email after oral invitation, obtaining the informed consent of the participants in paper form. Besides, the experiment was conducted anonymously and coded in the format of “surname-gender-title.” For example, “Wu-F-Prof.” represents a female professor with the last name Wu.

### 2.2. Material

The purpose of this study was to develop core competencies and a professional curriculum for care services to be taught at the vocational high school level in Taiwan. The research design covered three stages: content establishment, expert opinion collection, and expert review. The research tools included the Core Competency and Professional Syllabus Correspondence Table for Care Service Education in Vocational High Schools (Draft), Focus Group Interview Outline, and Delphi Questionnaire.

#### 2.2.1. Core Competency and Professional Syllabus Correspondence Table for Care Service Education in Vocational High Schools (Draft)

The research team reviewed relevant literature and referenced the 2019 Home Economics Syllabus for Vocational High Schools (Draft), the Test Specifications for Care Services of the Council of Labor Affairs, Executive Yuan, the professional curricula used in long-term care undergraduate programs, and the professional curricula used in the pilot care service departments of vocational high schools to formulate the Core Competency and Professional Syllabus Correspondence Table for Care Service Education in Vocational High Schools (Draft). The core competencies were based on the fundamental belief of the Curriculum Guidelines of the 12-Year Basic Education that education is based on the spirit of holistic education and the cultivation of practical professionals to meet industry demands. Therefore, core competencies can be divided into general and professional competencies. General competencies are based on the fundamental principles of “taking initiative, engaging in interaction, and seeking the common good” stipulated in the Curriculum Guidelines of the 12-Year Basic Education. Professional competencies are based on the skill indices and professional competencies required for development at the high school level stipulated in the “Test Specifications for Care Services.” The two dimensions of competencies comprised a total of seven constructs and 13 indicators, of which general competencies accounted for two constructs and four indicators and professional competencies accounted for five constructs and nine indicators. Subsequently, a professional curriculum comprising 25 subjects was developed based on the core competencies selected in this study (Please see Table A1).

#### 2.2.2. Focus Group Interview Outline

The objective of the proposed draft was to solicit a wide range of expert opinions in long-term care. Therefore, a five-item focus group interview outline was created. The items were the following: (1) What are your opinions of the two general competencies and five professional competencies selected for this draft? (2) What are your opinions on the three subjects (Introduction to Long-Term Care, Basic Care Practices and Experiments, and Practical Training on Basic Care) designed for the professional curriculum based on the Test Specifications for Long-Term Caregivers in Taiwan? (3) What are your opinions on the four subjects centered on the general competency indicators? (4) What are your opinions on the 25 professional subjects centered on the professional competency indicators selected for the draft? and (5) What are your recommendations for schools aspiring to develop unique teaching plans for long-term care?

#### 2.2.3. Delphi Questionnaire

The initial draft was modified based on the content analysis results in Stage 1 and the focus group interview in Stage 2. The first Delphi questionnaire was then compiled. To help the expert panel better understand the content of the questionnaire and provide answers, the questionnaire was accompanied by a research explanation letter and a set of instructions.

The purpose of the research explanation letter was to communicate relevant matters concerning the research with the experts. The research explanation letter for the first questionnaire included detailed research objectives, the implementation method and procedures, the expected schedule, and a glossary of terms. The research explanation letter for the second questionnaire and later questionnaires included the data processing method and results of the previous iteration, a compilation of expert opinions, and a list of amendments made in the current version of the questionnaire. The instructions presented the structure of the questionnaire and instructions on how to answer it. In the second questionnaire and later versions, a statistical analysis of the items in the previous iterations and a compilation of expert opinions were provided to serve as references when reconsidering the questionnaire items. The experts were asked to elaborate if their opinions differed from the overall consensus.

The questionnaire content was presented in three parts, specifically, a core competency survey, a professional curriculum survey, and other suggestions. The core competency survey comprised 21 items in two constructs. The questionnaire was scored on a 5-point scale, where 1 indicated “nonapplicable” and 5 indicated “very applicable.” The professional curriculum survey comprised 26 items in two constructs. This questionnaire was also scored on a 5-point scale, where 1 indicated “nonapplicable” and 5 indicated “very applicable.” The other suggestions section comprised an open-ended question in response to which experts could provide their suggestions regarding the current questionnaire. Thereafter, the researchers systematically analyzed the outcomes of the previous surveys and questionnaire content and considered the significant evaluation results and expert opinions for each item to produce subsequent iterations of the questionnaire.

### 2.3. Procedure

**Figure 1:**
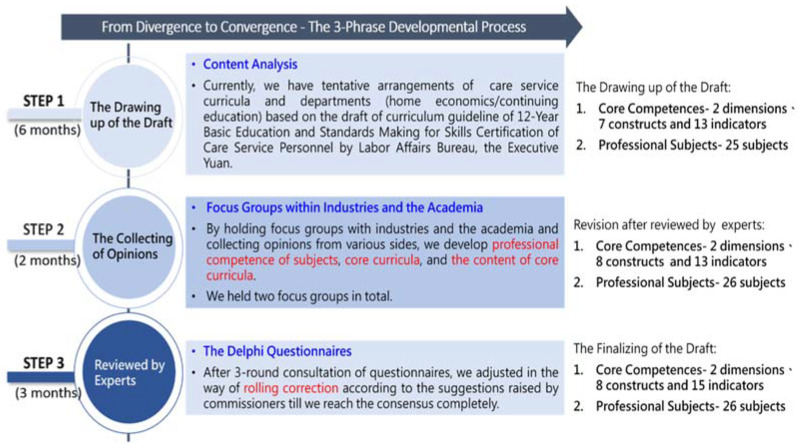
The Graph of Research Procedures.

The K-12 Education Administration, Ministry of Education, entrusted the current research team with identifying the core professional competencies and formulating a professional curriculum for the care service departments in vocational high schools in Taiwan. This study was conducted in three stages (Figure 1). The first stage comprised a literature review and content analysis, which took half a year. During the conceptualization of the proposed draft, we assembled a curriculum promotion task force comprising eight professors specializing in long-term care (including members involved in designing high school syllabuses for the Ministry of Education) to identify the core competencies and draft a professional curriculum. To collect more opinions, we organized two academic and industry focus groups during the second stage. The opinions of 20 experts and scholars of long-term care were collected. Each focus group continued until data saturation, lasting approximately 2 h. Two focus groups are eventually held in this research. During this phrase, it took 2 months to invite experts, organize focus groups, and analyze the data. In the third stage, the research results obtained in the first and second stages were consolidated and used to modify the draft. Thereafter, the modified draft was submitted for expert review. The Delphi survey method was adopted in the third stage and comprised three rounds of questionnaire surveys. The survey period was 3 months and involved a panel of 10 experts. The overall questionnaire recovery rate was 100%. Research was conducted as a team. The main research tasks included collecting and amending the research data, inviting and confirming the research subjects, and collecting and analyzing the research data. After each round of data collection and analysis, the research team encoded the data for data analysis and triangulation. This approach facilitated the research team attaining consensus and ensured reliability and validity.

The research can be exempt from the ethical review of human research according to the regulations of the Review Committee since the case does not take non-adults, inmates, aboriginals, pregnant women, the physically or mentally challenged, psychosis patients, or people under unreasonable threat or unable to decide based on free will as objects of research, nor conduct anonymous non-intrusive research in public places, and the information gathered is unrecognizable as specifically individual, and thus is exempt from the ethical review of the Review Committee (Ministry of Health and Welfare, 2012). The research also abides by the regulations of journals for non-interventional studies (e.g., surveys, questionnaires, social media research); all participants must be fully informed that anonymity is assured, why the research is being conducted, how their data will be used and if there are any risks associated. In this study, informed consent was obtained from all the participants on the basis of the aforementioned article, and because the research is commissioned by the public sectors for professional institutes in order to conduct national policy related research, the objects of research are for the purpose of sharing academic professional knowledge and providing recommendations, which do not belong to the domain governed by the Review Committee and thus can be exempt from ethical review. In order to make the participants better understand the research goal and comply with research ethics, we will send descriptions of the research first via email after oral invitation, obtaining the informed consent of the participants in paper form.

### 2.4. Data Analysis

A hierarchical stepwise induction approach was adopted for the focus groups. The researchers first highlighted the meaningful codes in the interview transcripts. Related, similar, or identical codes were grouped into units, and the units were consolidated into subthemes (Table 1). Finally, the researchers reviewed the themes to ensure that they conformed with the research objectives. To ensure objectivity, the data were cross-coded and cross-inspected within the research team. Subjective biases were avoided by engaging in collaborative discussions and interpretive dialogues or submitting the transcripts for peer review and discussion.

The survey comprised two parts, a qualitative analysis and a quantitative analysis. In the qualitative analysis, the researchers consolidated similar or identical opinions provided by the experts. The research team then discussed the opinions and determined whether adjustments were required. In the quantitative analysis, the responses to the three rounds of questionnaire surveys were presented as mean values and modes. The expert opinions and mean, standard deviation, and quartile deviation values were adopted as a reference for consistency and consensus [15]. To ensure the accuracy of the research results, the researchers adopted mean values, modes, quartile deviation values, and standard deviation values as the determination criteria. The reference criteria were as follows: (1) a mean value of ≥3.5 (1 to 5) or a mode value of ≥3 denoted a high level of importance, (2) a quartile deviation value of <1 or a standard deviation value of ≤1 denoted that expert consensus had been reached, and (3) a mean value of <3.5 (1 to 5) or a mode value of ≤3 and a quartile deviation value of <1 denoted deletion criteria.

## 3. Results

### 3.1. Analysis of the Focus Group Interview

In this study, we organized focus groups to determine the suitability of the research content and identify the key factors associated with the core competencies and professional curriculum of care services in high schools. The researchers modified the Core Competency and Professional Syllabus Correspondence Table for Care Service Education in Vocational High Schools based on the focus group results. Thereafter, the Delphi questionnaire was administered. Based on the focus group outcomes, first of all we added one construct to general competencies and modified “communication and care” to “interpersonal communication” and “care.” Originally, they were “Communication and Caring Competency”, along with “Creativity and Planning Capability”, but the members of the focus group consider that care, for caretakers, is an important capability; therefore, it is especially listed as an independent core competence and we hope that the capability can reach the indicator of “Creating shared value”; by means of care, the mutual goal can be obtained of common good between caretakers and care recipients. Therefore, the general competency dimension comprised three constructs (i.e., interpersonal communication, care, and creativity and execution) and four indicators. The five constructs in professional competencies were maintained. However, we considered that students may not yet have developed leadership skills in high school and replaced “leadership” with “problem-solving and improvisation.” Besides, two indicators were developed, the ability to analyze problems in case studies and the ability to solve problems in case studies, in the dimension of problem-solving and improvisation, with a view to cultivating among students professional capabilities to analyze and solve the care problems in specific cases. In the section “The Capability of Resource Integration”, we originally hoped that students could reach the indicator of “Getting to Know Related Laws and Regulations of Care Service”; however, after the assessment of committee members, they considered that the laws and regulations during high school are too difficult; moreover, high school graduates are usually frontline practitioners, so it was advised that the two indicators should be changed into “Being acquainted and connected with care resources” and “Encouraging health promotion activities”, hoping to connect resources and put this into practice. A total of 11 indicators were identified for the five constructs. The focus group identified 14 indicators across eight constructs in the general and professional competencies dimensions. (Change—the comparison, Table 2).

For the professional curriculum, three compulsory courses were identified for the caregiver examination, namely “Introduction to Long-Term Care”, “Basic Care Practices and Experiments”, and “Practical Training in Basic Care.” Other courses included institutional care, community care, and home care courses to foster situational care competency among students. Based on the revised capability, we deleted two curricula: “Hospice Care” and “An Introduction to Family”. Palliative care is an advanced course unsuitable for high school students. Therefore, this course was omitted. A “case management course” was added to coincide with the addition of the new core competency, “problem-solving and improvisation.” Besides, we added the “Introduction to Case Management” course, integrating “The Practice of Health Promotion” and “Community Health Construction I II” as “Practical Project (I) (II)”, appended “The Policies and Laws of Long-Term Care” and “Community Health Building”, and, as for the rest, some are adjustments in the names of the courses. Based on the expert recommendations, a total of 26 professional courses were identified in the focus group (Table 2).

### 3.2. Establishment and Analysis of the Core Competencies for Care Service Education in Vocational High Schools

A total of 14 indicators across eight constructs in two core competency dimensions were preliminarily identified based on the focus group outcomes. The core competencies were included in the first round of the Delphi questionnaire survey. After the questionnaires were recovered, the opinions provided by the expert panel were collected and analyzed. The original list of 14 indicators across eight constructs in two core competency dimensions was retained. However, the descriptions of “creativity and execution” and “resource connection and application” were modified. Committee members considered that students of high school age are mainly required to cultivate the competence of creativity activation and program execution; therefore, the original “spontaneous creation, program planning and execution” has been simplified as “spontaneous creation and program execution”. In addition, as for the capability to connect resources and put these into application, we added “to assist” to “to advertise health promotion activities”; for the main part, it is taken into consideration that students are not yet able to lead health promotion activities independently; as a result, we hope that we can first cultivate the capability to assist and promote activities of health promotion. Fourteen indicators across eight constructs in two core competency dimensions were included in the second round of the Delphi questionnaire survey. The opinions of the expert panel were once again collected and analyzed. The eight constructs and two dimensions were retained. However, one indicator was added for a total of 15 indicators, and the description of one indicator was modified. One indicator was added to the “problem-solving and improvisation” construct, and the original description was modified. We added “The Capability to Perceive the Problems of Cases” and changed “The Capability to Solve Problems” into “The Executive Capability to Solve Problems of Cases”, hoping to cultivate among students capabilities to discover and solve problems. The revised list of core competencies was carried over to the third round of the Delphi questionnaire survey. The experts provided no further opinions in the third round, and the statistical data met all of the pre-established criteria, suggesting that expert consensus was achieved. In this round, all the written expert opinions were acknowledged and corrected. The modifications made in the three rounds of the Delphi questionnaire survey are noted in Table 3.

The opinions provided by the expert panel in the three-round questionnaire survey concerning the overall importance and consistency of the core competencies of care services for education in vocational high schools are presented in Table 4. In the “general competencies” dimension, (1) the mean values for the “interpersonal communication” construct and relevant indicators ranged between 4.8 and 4.9. The mode value was 5, quartile deviation value was 0, and standard deviation value was less than 1. (2) The mean values for the “care” construct and relevant indicators ranged between 4.7 and 4.8. The mode value was 5, quartile deviation value was 0, and standard deviation value was less than 1. (3) The mean value for the “creativity and execution” construct and relevant indicators was 4. The mode value was 5, quartile deviation value was 0, and standard deviation value was less than 1. These results indicated that all the constructs and indicators in the general competencies were important and that the experts had a strong consensus regarding consistency.

In the “professional competencies” dimension, (1) the mean value for the “patient safety and care” construct and relevant indicators was 5. The mode value was 5, quartile deviation value was 0, and standard deviation value was less than 1. (2) The mean value for the “problem-solving and improvisation” construct and relevant indicators was 4.9. The mode value was 5, quartile deviation value was 0, and standard deviation value was less than 1. (3) The mean values for the “adherence to a professional code of conduct and ethics” construct and relevant indicators ranged between 4.9 and 5. The mode value was 5, quartile deviation value was 0, and standard deviation value was less than 1. These results indicated that all of the indicators in the “patient safety and care”, “problem-solving and improvisation”, and “adherence to a professional code of conduct and ethics” constructs were important and that the experts had a strong consensus regarding consistency. (4) The mean values for the “professional care” construct and relevant indicators ranged between 4.5 and 5. The mode value was 5, quartile deviation values were either 0 or 0.75, and standard deviation value was less than 1. These results indicated that this construct and its indicators were important and that the experts had a moderate-to-strong consensus regarding consistency. (5) The mean values for the “resource connection and application” construct and relevant indicators ranged between 4.4 and 4.6. The mode value was 5, quartile deviation values were either 0.75 or 1, and standard deviation value was less than 1. These results indicated that this construct and its indicators were important, but the experts had a weak consensus regarding consistency. An analysis of the expert opinions regarding the importance and consistency of the various constructs and indicators revealed that expert consensus was achieved. Overall, we developed two core competency dimensions containing 15 indicators across eight constructs for care service departments in vocational high schools.

### 3.3. Establishment and Analysis of a Professional Curriculum for Care Service Education in Vocational High Schools

A total of 26 subjects across eight constructs were preliminarily identified for the professional curriculum of care service education in vocational high schools based on a literature review and the focus group outcomes. This preliminary list of subjects and constructs was included in the first round of the Delphi questionnaire survey. After the questionnaires were recovered, the opinions provided by the expert panel were collected and analyzed. A total of 26 subjects were retained. However, one subject was modified in the “creativity and execution” construct. Committee members advised revision of “the application of assistive case design”, but they did not raise obvious suggestions for revision. Therefore, 26 subjects across eight constructs were included in the second round of the Delphi questionnaire survey. The opinions of the expert panel were again collected and analyzed. The 26 subjects and eight constructs were retained; however, one subject in the “problem-solving and improvisation” construct was modified. Committee members advised turning “Case Management” into “An Introduction to Case Management”; that is because the content of case management is too difficult for students at high school age, and they had only just attended the introductory course. The revised list of subjects and constructs was carried over to the third round of the Delphi questionnaire survey. The experts provided no further opinions in the third round, and the statistical data met all of the pre-established criteria, suggesting that expert consensus was achieved. In this round, all the written expert opinions were acknowledged and corrected. The modifications made in the three rounds of the Delphi questionnaire survey are presented in Table 5.

The opinions provided by the expert panel in the three-round questionnaire survey concerning the overall importance and consistency of the professional curriculum for care service education in vocational high schools are presented in Table 6. (1) In the “interpersonal communication” construct, the mean value was 4.9, mode value was 5, quartile deviation value was 0, and standard deviation value was less than 1. (2) In the “adherence to a professional code of conduct and ethics” construct, the mean value was 4.9, mode value was 5, quartile deviation value was 0, and standard deviation value was less than 1. These results indicated that all the subjects in the “interpersonal communication” and “adherence to a professional code of conduct and ethics” constructs were crucial, and the experts had a strong consensus regarding consistency. (3) In the “creativity and execution” construct, the mean values ranged between 4 and 4.4, mode values were either 4 or 5, quartile deviation values were either 0 or 1, and standard deviation value was less than 1. (4) In the “professional care” construct, the mean values ranged between 4.5 and 5, mode value was 5, quartile deviation values were either 0 or 0.75, and standard deviation value was less than 1. (5) In the “patient safety and care” construct, the mean values ranged between 4.6 and 5, mode value was 5, quartile deviation values were either 0 or 0.75, and standard deviation value was less than 1. (6) In the “resource connection and application” construct, the mean values ranged between 4.4 and 4.8, mode value was 5, quartile deviation values were either 0 or 1, and standard deviation value was less than 1. These results indicated that all of the subjects in the “creativity and execution”, “professional care”, “patient safety and care”, and “resource connection and application” constructs were vital or essential, and the experts had a strong consensus regarding the consistency of most of the subjects and a weak consensus regarding the consistency of a number of subjects. (7) In the “care” construct, the mean value was 4.7, mode value was 5, quartile deviation value was 0.75, and standard deviation value was less than 1. These results indicated that all of the subjects in this construct were important, but the experts had a weak consensus regarding their consistency. (8) In the “problem-solving and improvisation” construct, the mean values ranged between 4.2 and 4.8, mode values were either 4 or 5, quartile deviation values were either 0.25 or 1, and standard deviation value was less than 1. These results indicated that all of the subjects in this construct were essential. An analysis of the expert opinions regarding the importance and consistency of the various subjects and constructs for a professional curriculum revealed that expert consensus was achieved.

Overall, we developed 26 subjects for a professional care service curriculum for vocational high schools. One subject centered on “interpersonal communication”, one on “care”, two on “creativity and execution”, eight on “professional care”, seven on “patient safety and care”, three on "resource connection and application," two on “problem-solving and improvisation”, and two on “adherence to a professional code of conduct and ethics.” (For complete core competences of innovation and professional curricula, (please see Table A2).

## 4. Discussion 

Regarding the core competencies of care services, we identified 15 indicators across eight constructs: interpersonal communication, care, creativity and execution, professional care, patient safety and care, resource connection and application, problem-solving and improvisation, and adherence to a professional code of conduct and ethics. With social and economic development, long-term caregivers have gradually formed a consensus that “communication”, “assessment”, and “direct care” are the key skills required to provide long-term care to older adults [16,17,18,19]. A survey invited 363 nursing home workers and home caregivers with nursing backgrounds to vote on the abilities they deemed necessary for providing long-term care; the participants agreed that interpersonal communication and direct care were essential abilities [20]. The research proposed four crucial abilities of caregivers in nursing homes: (1) interpersonal skills, namely communication, motivation, and conflict management; (2) clinical skills, namely assessing care needs, preparing care plans, performing care tasks, and evaluating care performance; (3) intra-organizational skills, namely formulating plans and contingencies; and (4) management skills, namely following laws and regulations, planning financial budgets, and providing supervision and counseling [21]. The study proposed three major competencies: (1) interpersonal skills, namely communication, motivation, and conflict management; (2) organizational skills, namely organizational skills, planning service strategies, and utilizing resources; and (3) management skills, namely following laws and regulations, planning financial budgets, and providing supervision and counseling [22]. The conclusions of the preceding two studies are similar to that of the current study. However, when devising the core competencies for long-term care education in high schools, we considered that high school students have not yet developed management skills. Therefore, the development of resource connection and application rather than management abilities seemed more practical at the high school level. The American Geriatrics Society examined the trends of active aging and the establishment of age-friendly environments and recommended that professional undergraduate programs should focus on cultivating the ability to enhance health promotion and safety, assess care needs, formulate care plans and coordinate relevant services, work in a professional team, support caregivers, and utilize resources. These six constructs are similar to those proposed [23]. In summary, caregiver competencies directly influence care quality. Therefore, long-term caregivers must possess specific skills. When working in a team, the ability to work together, communicate with team members, comprehend organizational operations, and utilize resources are indispensable competencies. The number of older adults living alone or experiencing depression is rising in Taiwan. We considered these social trends and included “care” to be one of the eight core competencies to cultivate in professionals to address and improve the mental health of older adults.

In terms of the professional curriculum, we identified 26 subjects that constituted 15 core competency indicators and eight constructs for care service departments in vocational high schools. To facilitate caregiver attainment of national accreditation, professional care courses, such as Introduction to Long-Term Care I & II, Basic Care Practices and Experiments I & II, and Practical Training on Basic Care I & II, were listed as mandatory courses. These courses help caregivers attain their caregiver license after graduation and become eligible to work as front-line long-term caregivers. The training programs for cultivating long-term care professionals in Taiwan are offered at three levels, namely Level 1, Level 2, and Level 3. Level 1 programs focus on the fundamentals of long-term care. Subjects include an introduction to long-term care, long-term care requirements, care management, and introduction to and application of care resources [24]. Long-term care involves a wide range of factors. With the demographic structure becoming that of an aging society with a prevalence of chronic illnesses and increased complexities of long-term care, long-term care has evolved from provision of mainstream institutional care (hospitals and nursing homes) to community and home care. The conventional training method focusing on the disease care of older patients no longer meets current demands [25,26,27]. Therefore, we added three situational care constructs, “institutional care”, “home care”, and “community care”, to promote the development of flexible care skills in order to meet varied situational requirements in students. Furthermore, to help schools develop distinct long-term care characteristics and help students foster creativity, we included a thematic and practical course. Teachers can create thematic booths at high school science fairs and competitions with their students to help them foster the ability to formulate innovative care plans.

In Australia, a complete five-level long-term care vocational certificate (Certificate III) with corresponding eight-module courses has been developed to cultivate junior care attendants (https://tafeqld.edu.au/, 2019, accessed on 6 January 2022). Level 1 of the Aged care certificate is linked to Module course 1–2 (Orientation to aged care practice and skills & Assist with daily living); Level 2 of the Community care certificate is connected with Module course 6 (Promoting wellbeing); Level 3 of the Home care certificate corresponds to Module 3–4 (Assist with health issues & Communicating with the older person and family); Level 4 of the Disability care certificate is associated with Module Course 5 (Working with a person with a disability); Level 5 of the Advanced care certificate is related to Module Course 7–8 (Caring for the person at end stage of life & Caring for the person with dementia). On the other hand, six-module courses were designed in Japan for training junior long-term care personnel (http://www.care-manager.net/, 2009, accessed on 6 January 2022), including: (1). Introduction to long-term care personnel; (2). Care; (3). Rehabilitation; (4). Welfare and quality of life; (5). Dementia care; (6). Humanistic care training & social support etc. Comparing training courses in Australia and Japan, we found that Australia’s " Disability care " (Module Course 5) and “Advanced care” (Module Course 7 & 8), and Japan’s " Advanced care " are the main focus for development in Taiwan at the university level, but not included in the high school level. In the future, Taiwan can refer to the curriculum design of Australia and Japan, and consider the inclusion of training courses in related topics in high school. In particular, the current dementia population and the need for its special care in Taiwan are increasing, thus the related course should be worth introducing in our society.

## 5. Limitation and Further Studies

This study aimed to develop core competencies and a professional curriculum for care services to be taught at the vocational high school level in Taiwan. The research design covered three stages, content establishment, expert opinion collection, and expert review. This study establishes localized teaching guidelines that conform to Taiwanese culture, Taiwanese education norms, and high school students’ learning needs and learning levels. Therefore, the results of this study are limited to high school students and cannot be inferred to other age groups. This study did not follow up on the effectiveness of core competencies and the implementation of professional curriculum. It is recommended that related studies such as effectiveness evaluation be carried out in the future, when research can evaluate the effectiveness of teacher teaching and student learning, understand the effectiveness of its implementation in Taiwan and use this as a basis for subsequent revisions.

## 6. Implications

The rise in the demand for long-term care resulting from population aging has directed international attention to the need for long-term care. In the past, the core competencies required for providing long-term care could only be cultivated in universities. However, with the predicted exponential growth in the demand for long-term care in the next 10–15 years, the cultivation of long-term caregivers has become a critical concern. In response, the Taiwanese government has promoted the cultivation of caregivers in high school, encouraging vocational high schools to establish care service departments and learning pathways to meet long-term care demands in Taiwan, overturn the stereotype that long-term care is an unspecialized profession, and enhance the willingness of young adults to work in the long-term care industry. These efforts are currently in the preliminary stages. To foster students’ professional competencies, we identified the core competencies for long-term care service education and devised a professional curriculum to foster the skills and knowledge among students that are required for successfully meeting of the care needs of a rapidly aging society through work in the long-term care industry after graduation.

## 7. Conclusions

Regarding the core competencies of care services, we identified 15 indicators across eight constructs: interpersonal communication, care, creativity and execution, profession-al care, patient safety and care, resource connection and application, problem-solving and improvisation, and adherence to a professional code of conduct and ethics. In terms of the professional curriculum, we identified 26 subjects that constituted 15 core competency indicators and eight constructs for care service departments in vocation-al high schools. To facilitate caregiver attainment of national accreditation, professional care courses, such as Introduction to Long-Term Care I & II, Basic Care Practices and Ex-periments I & II, and Practical Training on Basic Care I & II, were listed as mandatory courses. These courses help caregivers attain their caregiver license after graduation and become eligible to work as front-line long-term caregivers. Furthermore, to help schools develop distinct long-term care characteristics and help students foster crea-tivity, we included a thematic and practical course. Teachers can create thematic booths at high school science fairs and competitions with their students to help them foster the abil-ity to formulate innovative care plans.

## Figures and Tables

**Table 1:** Example of a data analysis table.

Theme	Subtheme	Unit	Code
Future development of care service departments in vocational high schools	A comprehensive curriculum should consider optimal health, suboptimal health, and care requirement subjects	Course arrangement should be based on optimal health and suboptimal health courses while addressing disability	■ I stress that schools must play on their strengths, and course arrangement should be based on optimal health and suboptimal health courses while disability should be included in the other health courses. (Huang-F-P)
		Disability prevention courses, such as optimal health and suboptimal health, should be included	■ Development can be viewed in two dimensions. First, we should not focus on care. Second, we should include optimal health and suboptimal health groups and discuss “disability prevention” in our discussions. (Chen-M-N)
		Courses should cover healthy living and disease care	■ Care and healthy living were mentioned. I believe that these concepts should be included. Older adults must first lead a healthy lifestyle. They only require care when they are ill. As mentioned, these concepts must be emphasized. (Yang-M-P)

**Table 2:** Revision Reference Table of Core Competences and Professional Curriculum after Focus Group.

Core Competence (The Draft)	Core Competence (After Revisions)	Professional Subjects (After Revision)
**General Competency**		
**1. Communication and Caring Competency**	**1. Interpersonal communication**	⯎ Interpersonal relationships and communication
1.1 Interpersonal Relations and Communication Skills	1. 1 Interpersonal relationship	
1.2 Family Support: Needs and Assistance of Family Care	1. 2 Interactive literacy	
1.3 Hospice Care and getting to know hospice		
	**2. Care**	⯎ Introduction to human development
	2.1 Creating shared value	
**2. Creativity and Planning Capability**	**3. Creativity and execution**	⯎ Activity design and conduct ⯎ Design and applications for assistive program
2.1 The Planning of Gerontological Activity Programs	3.1 Proactive creativity and program execution	
**Professional Competency**		
**1. Professional care**	**1. Professional care**	
1.1 Basic Caring skills: Caring of the Body and Life; Dealing with Household Chores	1.1 Basic caring skills	⯎ **Introduction to long-term care (I)(II)** ⯎ **Practical basic care and experimental (I)(II)** ⯎ **Practicum for fundamental care (I)(II)**
1.2 Care in Context: Care Training of Different Categories	1.2 Practical caring skills in real situations	⯎ Long-term care facilities and practice techniques ⯎ Practicum for community care ⯎ Practicum for home care (I)(II)
1.3 General Clinical Nursing Techniques	1.3 Accuracy of case assessment ability	⯎ Structure and function of human body (I)(II) ⯎ Physical examination and assessment (I)(II)
**2. Patient safety and care**	**2. Patient safety and care**	
2.1 Dealing with accidents and emergencies	2.1 Dealing with accidents and emergencies	⯎ Dealing with accidents and emergencies ⯎ Home security and accident prevention
2.2 Care and life safety	2.2 Care and life safety	⯎ Introduction to nutrition (I)(II) ⯎ Disease prevention and chronic care ⯎ Introduction to drug therapy and medication safety ⯎ Assistive technology device and health care ⯎ Practicum for assistive technology device
**3. Adherence to a professional code of conduct and ethics**	**3. Adherence to a professional code of conduct and ethics**	
3.1 Abiding by Work Ethics	3.1 Abiding by Work Ethics	⯎ Ethics in long-term care
	3.2 Understanding the regulations related to caring services	⯎ Long-term care policy and regulation
**4.** **The Capability of Leadership**		
4.1 Developing Activities of Health Promotion		
4.2 Promoting Active Aging in Communities		
	**4. Problem-solving and improvisation**	⯎ Introduction to case management ⯎ Practical project (I)(II)
	4.1 Ability to analyze problems in case studies	
	4.2 Ability to solve problems in case studies	
**5. The Capability of Resource Integration**	**5. Resource connection and application**	⯎ Introduction to social welfare ⯎ Health promotion practicum ⯎ Community health building
5.1 Getting to Know Related Laws and Regulations of Care Service	5.1 Being acquainted and connected with care resources	
	5.2 Encouraging health promotion activities	

**Table 3:** Questionnaire amendments of the core competencies in the three-round Delphi questionnaire survey (*n* = 10).

Indicator Dimension	Indicator Construct	Amendments in Round 1					Amendments in Round 2					Amendments in Round 3					Final Items
		Original Items	Retained	Amended	Deleted	Added	Original Items	Retained	Amended	Deleted	Added	Original Items	Retained	Amended	Deleted	Added	
**General competencies**	**Interpersonal communication**	2	2	0	0	0	2	2	0	0	0	2	0	0	0	0	2
	**Care**	1	1	0	0	0	1	1	0	0	0	1	0	0	0	0	1
	**Creativity and execution**	1	1	1	0	0	1	1	0	0	0	1	0	0	0	0	1
**Professional competencies**	**Professional care**	3	3	0	0	0	3	3	0	0	0	3	0	0	0	0	3
	**Patient safety and care**	2	2	0	0	0	2	2	0	0	0	2	0	0	0	0	2
	**Resource connection and application**	2	2	1	0	0	2	2	0	0	0	2	0	0	0	0	2
	**Problem-solving and improvisation**	1	1	0	0	0	1	1	1	0	1	2	0	0	0	0	2
	**Adherence to a professional code of conduct and ethics**	2	2	0	0	0	2	2	0	0	0	2	0	0	0	0	2

**Table 4:** Overall importance and consistency of the core competencies for care service education in vocational high schools (*n* = 10).

Indicator Dimension	Indicator Construct and Indicators	Importance		Consistency	
		Mean	Mode	Quartile Deviation	Standard Deviation
**General competencies**	**1. Interpersonal communication**	**4.9**	**5**	**0**	**0.3**
	1. 1 Interpersonal relationship	4.9	5	0	0.3
	1. 2 Interactive literacy	4.8	5	0	0.4
	**2. Care**	**4.7**	**5**	**0**	**0.6**
	2.1 Creating shared value	4.8	5	0	0.6
	**3. Creativity and execution**	**4.0**	**4**	**0**	**0.4**
	3.1 Proactive creativity and program execution	4.0	4	0	0.4
**Professional competencies**	**4. Professional care**	**4.9**	**5**	**0**	**0.3**
	4.1 Basic caring skills	5.0	5	0	0
	4.2 Practical caring skills in real situations	4.5	5	0	0.7
	4.3 Accuracy of case assessment ability	4.5	5	0.75	0.9
	**5. Patient safety and care**	**5.0**	**5**	**0**	**0**
	5.1 Dealing with accidents and emergencies	5.0	5	0	0
	5.2 Care and life safety	5.0	5	0	0
	**6. Resource connection and application**	**4.6**	**5**	**0.75**	**0.7**
	6.1 Being acquainted and connected with care resources	4.6	5	0.75	0.7
	6.2 Encouraging health promotion activities	4.4	5	1	0.8
	**7. Problem-solving and improvisation**	**4.9**	**5**	**0**	**0.3**
	7.1 Ability to analyze problems in case studies	4.9	5	0	0.3
	7.2 Ability to solve problems in case studies	4.9	5	0	0.3
	**8. Adherence to a professional code of conduct and ethics**	**5.0**	**5**	**0**	**0**
	8.1 Compliance with the code of ethics and regulations	5.0	5	0	0
	8.2 Understanding the regulations related to caring services	4.9	5	0	0.3

**Table 5:** Questionnaire amendments of the professional curriculum in the three-round Delphi questionnaire survey (*n* = 10).

Indicator	Amendments in Round 1					Amendments in Round 2					Amendments in Round 3					Final Items
	Original Items	Retained	Amended	Deleted	Added	Original Items	Retained	Amended	Deleted	Added	Original Items	Retained	Amended	Deleted	Added	
**1. Interpersonal communication**																
1.1 Interpersonal relationship 1.2 Interactive literacy	1	1	0	0	0	1	1	0	0	0	1	1	0	0	0	1
**2. Care**																
2.1 Creating shared value	1	1	0	0	0	1	1	0	0	0	1	1	0	0	0	1
**3. Creativity and execution**																
3.1 Proactive creativity and program execution	2	1	1	0	0	2	2	0	0	0	2	2	0	0	0	2
**4. Professional care**																
4.1 Basic caring skills	3	3	0	0	0	3	3	0	0	0	3	3	0	0	0	3
4.2 Practical caring skills in real situations	3	3	0	0	0	3	3	0	0	0	3	3	0	0	0	3
4.3 Accuracy of case assessment ability	2	2	0	0	0	2	2	0	0	0	2	2	0	0	0	2
**5. Patient safety and care**																
5.1 Dealing with accidents and emergencies	2	2	0	0	0	2	2	0	0	0	2	2	0	0	0	2
5.2 Care and life safety	5	5	0	0	0	5	5	0	0	0	5	5	0	0	0	5
**6. Resource connection and application**																
6.1 Being acquainted and connected with care resources 6.2 Encouraging health promotion activities	3	3	0	0	0	3	3	0	0	0	3	3	0	0	0	3
**7. Problem-solving and improvisation**																
7.1 Ability to analyze problems in case studies 7.2 Ability to solve problems in case studies	2	2	0	0	0	2	1	1	0	0	2	2	0	0	0	2
**8. Adherence to a professional code of conduct and ethics**																
8.1 Compliance with the code of ethics and regulations 8.2 Understanding the regulations related to caring services	2	2	0	0	0	2	2	0	0	0	2	2	0	0	0	2

**Table 6:** Overall importance and consistency of the professional curriculum for care service education in vocational high schools (*n* = 10).

Indicator Dimension	Indicator Construct and Indicators	Professional Subjects	Importance		Consistency	
			Mean	Mode	Quartile Deviation	Standard Deviation
**General competency**	**1. Interpersonal communication**					
	1.1 Interpersonal relationship 1.2 Interactive literacy	Interpersonal relationships and communication	4.9	5	0	0.3
	**2. Care**					
	2.1 Creating shared value	Introduction to human development	4.7	5	0.75	0.5
	**3. Creativity and execution**					
	3.1 Proactive creativity and program execution	Activity design and conduct	4.4	5	1	0.8
		Design and applications for assistive program	4.0	4	0	0.6
**Professional competency**	**4. Professional care**					
	4.1 Basic caring skills	Introduction to long-term care (I)(II)	5.0	5	0	0
		Practical basic care and experimental (I)(II)	4.9	5	0	0.3
		Practicum for fundamental care (I)(II)	5.0	5	0	0
	4.2 Practical caring skills in real situations	Long-term care facilities and practice techniques	4.8	5	0	0.6
		Practicum for community care	4.7	5	0	0.6
		Practicum for home care (I)(II)	4.7	5	0	0.6
	4.3 Accuracy of case assessment ability	Structure and function of human body (I)(II)	4.7	5	0	0.6
		Physical examination and assessment (I)(II)	4.5	5	0.75	0.8
	**5. Patient safety and care**					
	5.1 Dealing with accidents and emergencies	Dealing with accidents and emergencies	5.0	5	0	0
		Home security and accident prevention	5.0	5	0	0
	5.2 Care and life safety	Introduction to nutrition (I)(II)	4.8	5	0	0.6
		Disease prevention and chronic care	4.7	5	0	0.6
		Introduction to drug therapy and medication safety	4.6	5	0.75	0.7
		Assistive technology device and health care	4.7	5	0	0.6
		Practicum for assistive technology device	4.8	5	0	0.6
	**6. Resource connection and application**					
	6.1 Being acquainted and connected with care resources 6.2 Encouraging health promotion activities	Introduction to social welfare	4.7	5	0	0.6
		Health promotion practicum	4.8	5	0	0.4
		Community health building	4.4	5	1	0.8
	**7. Problem-solving and improvisation**					
	7.1 Ability to analyze problems in case studies 7.2 Ability to solve problems in case studies	Introduction to case management	4.8	5	0.25	0.4
		Practical project (I)(II)	4.2	4	1	0.7
	**8. Adherence to a professional code of conduct and ethics**					
	8.1 Compliance with the code of ethics and regulations 8.2 Understanding the regulations related to caring services	Ethics in long-term care	4.9	5	0	0.3
		Long-term care policy and regulation	4.9	5	0	0.3

## Data Availability

The datasets used and analyzed in this study are available from the corresponding author upon reasonable request.

## Appendix A

**Table A1:** Core Competency and Professional Syllabus Correspondence Table for Care Service Education in Vocational High Schools (Draft).

General Competency	Professional Competency	Corresponding Subjects	
		The Names of Subjects	Credits
**1. Communication and Caring Competency**			
1.1 Interpersonal Relations and Communication Skills		⯎ Interpersonal Relations and Communication	2
1.2 Family Support: Needs and Assistance of Family Care		⯎ An Introduction to Family (Family Pressures and Family Dynamic are required to be included.)	2
1.3 Hospice Care and getting to know hospice		⯎ Hospice Care	2
**2. Creativity and Planning Capability**			
2.1 The Planning of Gerontological Activity Programs		⯎ The Design of Gerontological Activity ⯎ The Design and Application of Alternative Caring Programs	2 4
	**1.** **Professional care**		
	1.1 Basic Caring skills: Caring of the Body and Life; Dealing with Household Chores	⯎ Introduction to long-term care (I)(II) ⯎ Practical basic care and experimental (I)(II) ⯎ Practicum for fundamental care (I)(II)	4 4 4
	1.2 Care in Context: Care Training of Different Categories	⯎ The Practice of Care in Institutions (I)(II) ⯎ The Practice of Care in Communities (I)(II) ⯎ Dementia Care (I)(II)	4 4 4
	1.3 General Clinical Nursing Techniques	⯎ Structure and function of human body (I)(II) ⯎ Physical examination and assessment (I)(II)	4 4
	**2.** **Patient safety and care**		
	2.1 Dealing with Emergency and Accidental Events	⯎ Occupational Safety and Harm Prevention	2
	2.2 Care and life safety	⯎ Introduction to Nutrition (I)(II) ⯎ The Practice of Healthy Diet (I)(II) ⯎ The Prevention of Disease and the Care of Chronic Disease ⯎ Getting to Know the Medicine and the Safety of taking ⯎ Assistive technology device and health care(I)(II) ⯎ Practicum for assistive technology device (I)(II)	4 4 2 2 4 4
	**3. Adherence to a professional code of conduct and ethics**		
	3.1 Abiding by Work Ethics	⯎ The Professional Ethics of Long-Term Care	2
	**4. The Capability of Leadership**		
	4.1 Developing Activities of Health Promotion 4.2 Promoting Active Aging in Communities	⯎ The Practice of Health Promotion ⯎ Community Health Construction I II	2 4
	**5. The Capability of Resource Integration**		
	5.1 Getting to Know Related Laws and Regulations of Care Service	⯎ An Introduction to Long-Term Care Laws and Regulations ⯎ Health promotion practicum	2 2

**Table A2:** Core Competency and Professional Syllabus Correspondence Table for Care Service Education in Vocational High Schools.

General Competency	Professional Competency	Corresponding Subjects	
		The Names of Subjects	Credits
**1. Interpersonal communication**			
1.1 Interpersonal relationship 1.2 Interactive literacy		⯎ Interpersonal relationships and communication	2
**2. Care**			
2.1 Creating shared value		⯎ Introduction to human development	2
**3. Creativity and execution**			
3.1 Proactive creativity and program execution		⯎ Activity design and conduct ⯎ Design and applications for assistive program	2 2
	**1. Professional care**		
	1.1 Basic caring skills	⯎ Introduction to long-term care (I)(II) ⯎ Practical basic care and experimental (I)(II) ⯎ Practicum for fundamental care (I)(II)	4 4 4
	1.2 Practical caring skills in real situations	⯎ Long-term care facilities and practice techniques ⯎ Practicum for community care ⯎ Practicum for home care (I)(II)	4 4 4
	1.3 Accuracy of case assessment ability	⯎ Structure and function of human body (I)(II) ⯎ Physical examination and assessment (I)(II)	4 4
	**2. Patient safety and care**		
	2.1 Dealing with accidents and emergencies	⯎ Dealing with accidents and emergencies ⯎ Home security and accident prevention	2 2
	2.2 Care and life safety	⯎ Introduction to nutrition (I)(II) ⯎ Disease prevention and chronic care ⯎ Introduction to drug therapy and medication safety ⯎ Assistive technology device and health care ⯎ Practicum for assistive technology device	4 2 2 2 2
	**3. Resource connection and application**		
	3.1 Being acquainted and connected with care resources 3.2 Encouraging health promotion activities	⯎ Introduction to social welfare ⯎ Health promotion practicum ⯎ Community health building	2 2 2
	**4.** **Problem-solving and improvisation**		
	4.1 Ability to analyze problems in case studies 4.2 Ability to solve problems in case studies	⯎ Introduction to case management ⯎ Practical project (I)(II)	2 4
	**5. Adherence to a professional code of conduct and ethics**		
	5.1 Compliance with the code of ethics and regulations 5.2 Understanding the regulations related to caring services	⯎ Ethics in long-term care ⯎ Long-term care policy and regulation	2 2
